# FibrilPaint to determine the length of Tau amyloids in fluids

**DOI:** 10.1073/pnas.2502847122

**Published:** 2025-10-27

**Authors:** Júlia Aragonès Pedrola, Françoise A. Dekker, Tommaso Garfagnini, Guy Mayer, Margreet B. Koopman, Menno Bergmeijer, Gobert Heesink, Iris Rots, Mireille M. A. E. Claessens, Friedrich Förster, Jeroen J. M. Hoozemans, Henrik Jensen, Assaf Friedler, Stefan G. D. Rüdiger

**Affiliations:** ^a^Protein Chemistry of Disease, Department of Chemistry, Utrecht University, Utrecht 3584CH, The Netherlands; ^b^Cellular Protein Chemistry, Bijvoet Center for Biomolecular Research, Utrecht University, Utrecht 3584CH, The Netherlands; ^c^Science for Life, Utrecht University, Utrecht 3584CH, The Netherlands; ^d^Institute of Chemistry, The Hebrew University of Jerusalem, Edmond J. Safra Campus at Givat Ram, Jerusalem 9190401, Israel; ^e^Structural Biochemistry, Bijvoet Centre for Biomolecular Research, Utrecht University, Utrecht 3584 CG, The Netherlands; ^f^Nanobiophysics, Faculty of Science and Technology, MESA + Institute for Nanotechnology and Technical Medical Centre, University of Twente, Enschede 7500AE, The Netherlands; ^g^Department of Pathology, Amsterdam Neuroscience, Amsterdam UMC-location VUmc, Amsterdam 1081 HV, The Netherlands; ^h^Fida Biosystems ApS Generatorvej, Søborg 6 A+B 2860, Denmark

**Keywords:** protein aggregation, Tau, microfluidics

## Abstract

Tau aggregation into amyloid fibrils is linked to the development of neurodegenerative diseases, including Alzheimer’s disease (AD). The molecular processes driving aggregation in disease are still being uncovered, highlighting the need for innovative tools to study aggregation reactions. Here, we introduce FibrilPaint1 as a tool to measure the size of Tau amyloid fibrils in fluids, from early aggregation stages to mature fibrils. FibrilPaint1 is a 22mer peptide with exciting properties: i) FibrilPaint1 binds fibrils with nanomolar affinity; ii) it also binds to precursors, down to a size of only 4 layers; iii) it does not bind to monomers; iv) it is fluorescently labeled, which allows monitoring and localizing interactions; v) FibrilPaint1 recognizes various Tau fibrils, including patient-derived fibrils from AD, corticobasal degeneration (CBD), and frontotemporal dementia (FTD); vi) it also binds to fibrils from amyloids derived from Amyloid-β, α-synuclein, and huntingtin vii) FibrilPaint1 is selective for the amyloid state and does not have background binding to amorphous aggregates, blood serum, or cell lysate. In combination with flow-induced dispersion analysis (FIDA), a microfluidics technology, we determined the molecular size of amyloid fibrils with submicroliter sample volumes. This setup acts as a molecular ruler at layer resolution—we determined Tau fibril length from 4 to 1100 layers in solution. This is an interesting parameter for molecular studies in dementia, with potential for diagnostic applications.

## Significance

The Tau protein forms amyloid fibrils in diseases like Alzheimer’s. Development of Tau fibrils correlate with disease progression. Until now, it has been very difficult to measure the size of these fibrils directly in solution. We developed the FibrilRuler Test to precisely measure fibril length in microliter sample volumes, acting as a molecular ruler. This enables the determination of Tau fibril size even at low nanomolar concentrations, allowing the study of fibril length modulation directly in solution. Such measurements offer valuable insights into how compounds or biological processes influence amyloid fibril elongation or fragmentation. Potentially, the FibrilRuler test may also be transformed into a diagnostic test to directly determine the size of amyloid fibrils in patient-derived material.

The aggregation of proteins into amyloid fibrils characterizes the development of neurodegenerative diseases, that affect 55 million people worldwide (1, 2). Amyloids are long, fibrillar structures with a typical cross-β fold (3–5). Amyloid formation of the protein Tau is related to the progression of several Tauopathies, including Alzheimer’s disease (AD), frontotemporal dementia (FTD), and corticobasal degeneration (CBD) (6–8). Tau is an intrinsically disordered protein that can aggregate into distinct conformations when detached from the axons of neurons (4, 8–11).

The formation of amyloid fibrils is one of the earliest pathological hallmarks of neurodegenerative diseases (3, 6, 12–15). Recently, the antibodies Aducanumab and Lecanemab have been approved to target Amyloid-β (Aβ) plaques in the brain, aiming to slow down the progression of the disease and improve cognitive function (16, 17). In AD, Aβ aggregation outside the cell precedes Tau aggregation inside the cell (4, 13, 18). The effects of these antibodies remain modest, with significant risks involved, and most patients are not eligible for these therapies, as they apply only to very early stages of the disease (16, 17, 19, 20). Future therapies and treatments would strongly benefit if they could be administered early, preferentially before the first symptoms appear (18, 21–23).

Molecular understanding of aggregation is crucial for the development of new causal therapeutic strategies. Key for this is a quantitative understanding of the underlying nucleation and elongation phases of the process (24, 25). Nucleation is the formation of a seed that starts the aggregation process (26, 27). Such nuclei that initiate the aggregation reaction already form during a lag phase populated by transient and highly dynamic states of the protein, although nucleation events can continue beyond this phase. Elongation is the stacking of fibril layers on top of each other, lengthening the fibrils (25, 27). During secondary nucleation, existing fibrils act as catalytic surfaces that promote the formation of new nuclei from soluble monomers.

Accurate measurement of these steps remains a challenge. Small amyloid fibrils among these first nucleation steps easily escape detection, although already present in the lag phase (28, 29). Elongation remains challenging to characterize accurately. Amyloid dyes can estimate the presence of amyloids, but they cannot provide information on the size of the amyloid fibrils (30–33). Microscopy techniques visualize fibril structures down to atomic resolution, but they are limited in scalability and quantitative readout. To reveal a quantitative picture of the aggregation process, it is desirable to develop new sensitive and quantitative microscale methods in solution.

Here, we present a method to measure the length of Tau amyloid fibrils in solution. We developed FibrilPaint1, a peptide that specifically binds to Tau amyloid fibrils, but not monomers. In our FirbilRuler Assay, we combine FibrilPaint1 with flow-induced dispersion analysis (FIDA), to determine the fibril length of these Tau fibrils in fluids, such as blood plasma. We show its applicability to follow the elongation of recombinant TauRD, the repeat domain of Tau protein, over time. We also used this setup to determine the length of end-stage Tau fibrils from patients diagnosed with AD, FTD, and CBD. Finally, we show the specificity of FibrilPaint1 for Aβ42, α-Syn, and HttEx1Q44 amyloid fibrils, which are unrelated in structure and sequence. Together, this indicates that FibrilPaint1 is an amyloid-specific binder.

## Results

### Development of a Method to Detect Tau Amyloids in Fluids.

We set out to develop a method to detect Tau amyloid fibrils. To do so, we need a specific tracer to bind these Tau fibrils. Based on our previous work (34), we designed a set of four peptides with enhanced binding to amyloid aggregates. We increased the π-stacking and H-bonding residues of the peptides (34, 35). In addition, we added a negatively charged EEVD sequence C-terminally with a GSGS spacer, creating two oppositely charged sections, with a positively charged N-terminal region and a negatively charged C-terminus. The EEVD sequence may also serve as an adaptor to bind protein quality control factors that specifically recognize this sequence via a TPR domain, which is potentially interesting for follow-up studies (36, 37). We added a fluorescein group (Fl-) at the N terminus to allow detection. We varied the net charge and the number of aromatics in the peptides. This further optimization resulted in a set of four 22-residue-long peptides with different charge, number of aromatics, and distribution of the different types of residues (*SI Appendix*, Table S1). Designed to make fibrils visible, we call this class of peptides FibrilPaint. FibrilPaint1 and FibrilPaint2 have Arginine residues on positions 4 and 5, whereas FibrilPaint3 and FibrilPaint4 have Aspartates. FibrilPaint1 and FibrilPaint4 have Histidines on positions 11 and 12, where FibrilPaint2 and FibrilPaint3 have Threonines.

### The Four Peptides Interact With Tau Fibrils.

**Fig. 1.**
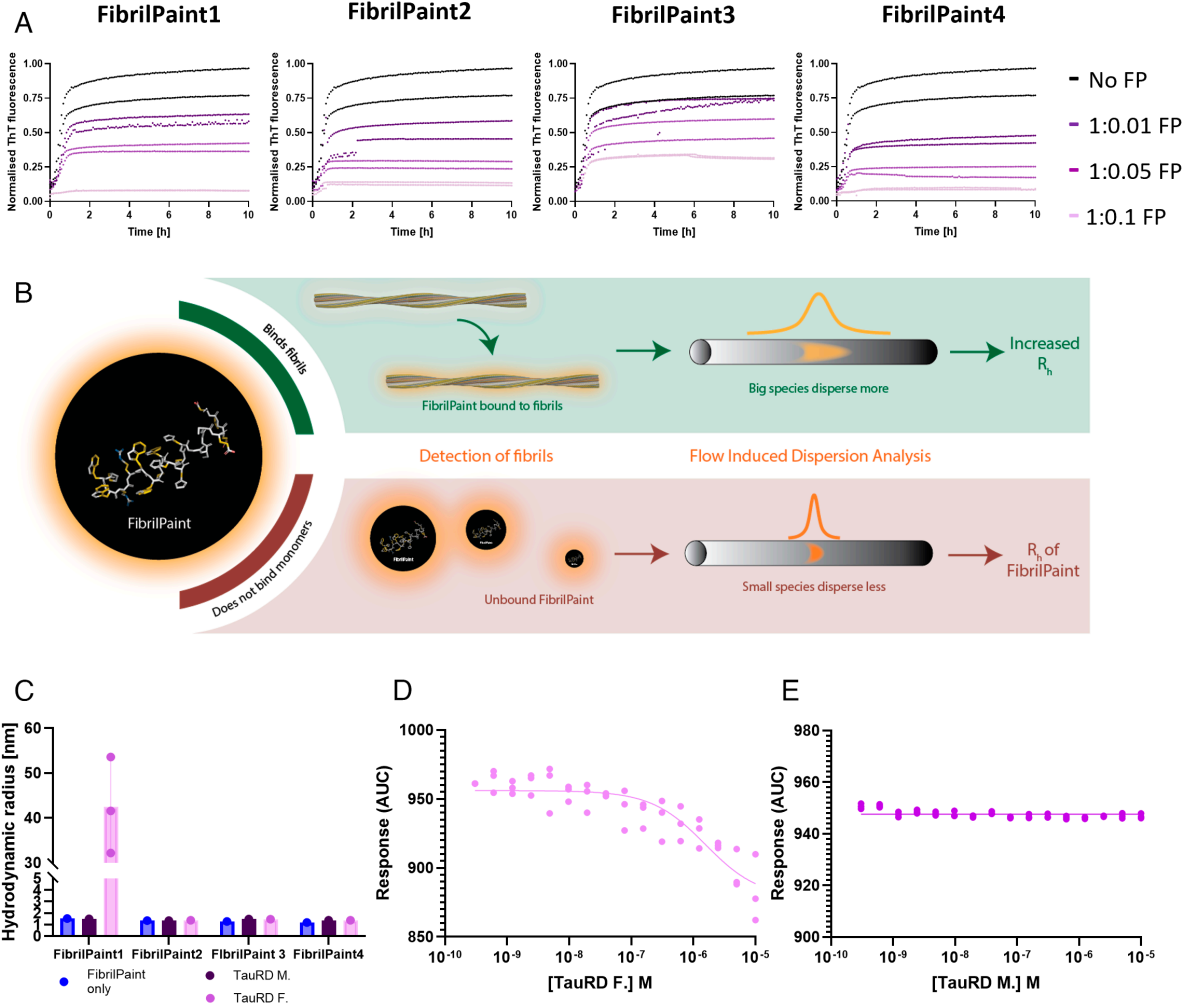
FibrilPaint1 is a specific label for protein fibrils. (*A*) Representative figure of ThT assays with 20 μM TauRD aggregation and titration of 2 to 0.2 to 0.02 µM FibrilPaint1, FibrilPaint2, FibrilPaint3, or FibrilPaint4 (*Left* to *Right*). All peptides lower the end-plateau in a dose-dependent manner. Triplicates are shown. N = 3 (*B*) Schematic of FibrilPaint binding to fibrils with Flow Induced Dispersion Analysis (FIDA). If a FibrilPaint binds, this leads to a bigger species, which disperses more in FIDA, leading to an increase in the perceived R_h_ (green box). If there is no binding, the R_h_ remains the same as the R_h_ of FibrilPaint only (red box). (*C*) Results FIDA of FibrilPaint binding to monomeric or fibrillar TauRD. Average of N = 3 shown. (*D*) Binding curve of FibrilPaint1 with titrated TauRD fibrils (N = 3, K_d_ 1.6E−6), or (*E*) monomers N = 3 (N = 3, K_d_ > 1E−5). (FibrilPaint peptide only, blue; TauRD monomers, dark purple; TauRD fibrils, light purple).

We used Tau Repeat Domain (Q244-E372, TauRD) with the proaggregation mutation ΔK280 as a model for screening our FibrilPaint compounds. We performed a Thioflavin T (ThT) assay. ThT is an amyloid dye and provides a semiqualitative method used for the identification of amyloid structures in vitro and histology staining (32, 33, 38). A ThT assay is an established method to monitor protein fibrils formation, in which the fluorescent dye ThT emits fluorescence upon binding to fibrils (38). We identified 45 µM ThT as optimal concentration to study the kinetics of TauRD aggregation (*SI Appendix*, Fig. S1), in line with the literature (39). The four FibrilPaint peptides decreased the emitted ThT signal significantly, at substoichiometric concentration in a dose-dependent manner (Fig. 1*A*). This demonstrates that the FibrilPaints interact with the TauRD fibrils.

### FibrilPaint1 Binds to Tau Fibrils.

Next, we screened the four FibrilPaint peptides for direct binding to TauRD fibrils. We developed an application of FIDA to determine the size of amyloid aggregates, called the FibrilRuler Test (Fig. 1*B*). FIDA is a recently established technique to measure size of protein complexes (40, 41). In FIDA, the detector records the fluorescence signal after passage of the sample through a long capillary. The sample passes a detector window, resulting in a size-dependent diffusion profile of the fluorescent compound. Smaller species diffuse faster and bigger ones slower, resulting in a narrow or broad dispersion of the fluorescent signal (Fig. 1*B*). Since FIDA readout relies on the physical diffusion properties of the sample, we can measure the hydrodynamic radius (R_h_) of labeled species from the fluorescent signal (42–44). The R_h_ is a parameter corresponding to the size and shape of a molecule or a complex in solution. It is the radius of the sphere created by the tumbling particle. R_h_ values can be estimated from the structural coordinates, either by experimental methods or by predictions such as AlphaFold (*SI Appendix*, Table S2). For known structures, e.g., for many protein fibrils, an experimentally determined R_h_ value allows us to estimate the dimensions of the particle.

With this setup, we determined the R_h_ of FibrilPaint1 to 4 to be 1.7 nm each (*SI Appendix*, Table S1). The binding of the fluorescently labeled FibrilPaint peptide to protein fibrils increases the R_h_ value, as the peptide now tumbles together with the larger fibril (Fig. 1*B*). To measure the R_h_ of fibril complexes, we incubated preformed fibrils together with each of the four peptides in a 1:100 peptide:monomer ratio, using 200 nM peptide with fibrils made of 20 μM TauRD monomers.

Of all the FibrilPaint peptides, only FibrilPaint1 bound to TauRD fibrils, increasing the average R_h_ value from 1.7 to 45 nm (Fig. 1*C*). Importantly, incubation of FibrilPaint1 with TauRD monomer did not result in an increased R_h_ value, indicating that it does bind specifically to the fibril but not to the monomer (Fig. 1*C*). It is interesting that only FibrilPaint1 binds strongly enough to act as a noncovalent label, given that all FibrilPaint peptides interact with TauRD fibrils during the aggregation process (Fig. 1*A*). This indicates that high affinity for amyloid fibrils exceeds the demands required for ThT competition or modulation of aggregation.

We confirmed that FibrilPaint1 binds specifically TauRD fibrils and not monomers by assessing binding affinity with monolith spectral shift analysis (Fig. 1 *D* and *E*). FibrilPaint1 shows no binding affinity for monomers (Fig. 1*E*) but does for TauRD amyloid fibrils (Fig. 1*D*). K_d_ in this fit is 1.6 µM, based on monomer concentration. Since many monomers are needed to form one fibril, true affinity lies higher.

The reduction in ThT fluorescence initially suggested that FibrilPaint1 might inhibit TauRD aggregation (Fig. 1*A*). To clarify this, we performed experiments where FibrilPaint1 was either present throughout the aggregation process or introduced only after aggregation was complete. When FibrilPaint1 was added after aggregation, the resulting fibrils were similar in size and morphology to those formed in its absence (*SI Appendix*, Fig. S2*A*). In contrast, the presence of FibrilPaint1 during aggregation prevented fibril formation, as confirmed by the absence of fibrils in TEM and by FIDA measurements showing only small particles (R_h_ ≈ 4 nm; *SI Appendix*, Fig. S2*B*). To ensure accurate size measurements, we therefore consistently added FibrilPaint1 after fibril formation at designated time points for FIDA analysis, so it does not interfere with the aggregation process.

### FibrilPaint1 Does Not Bind to Amorphous Aggregates.

**Fig. 2.**
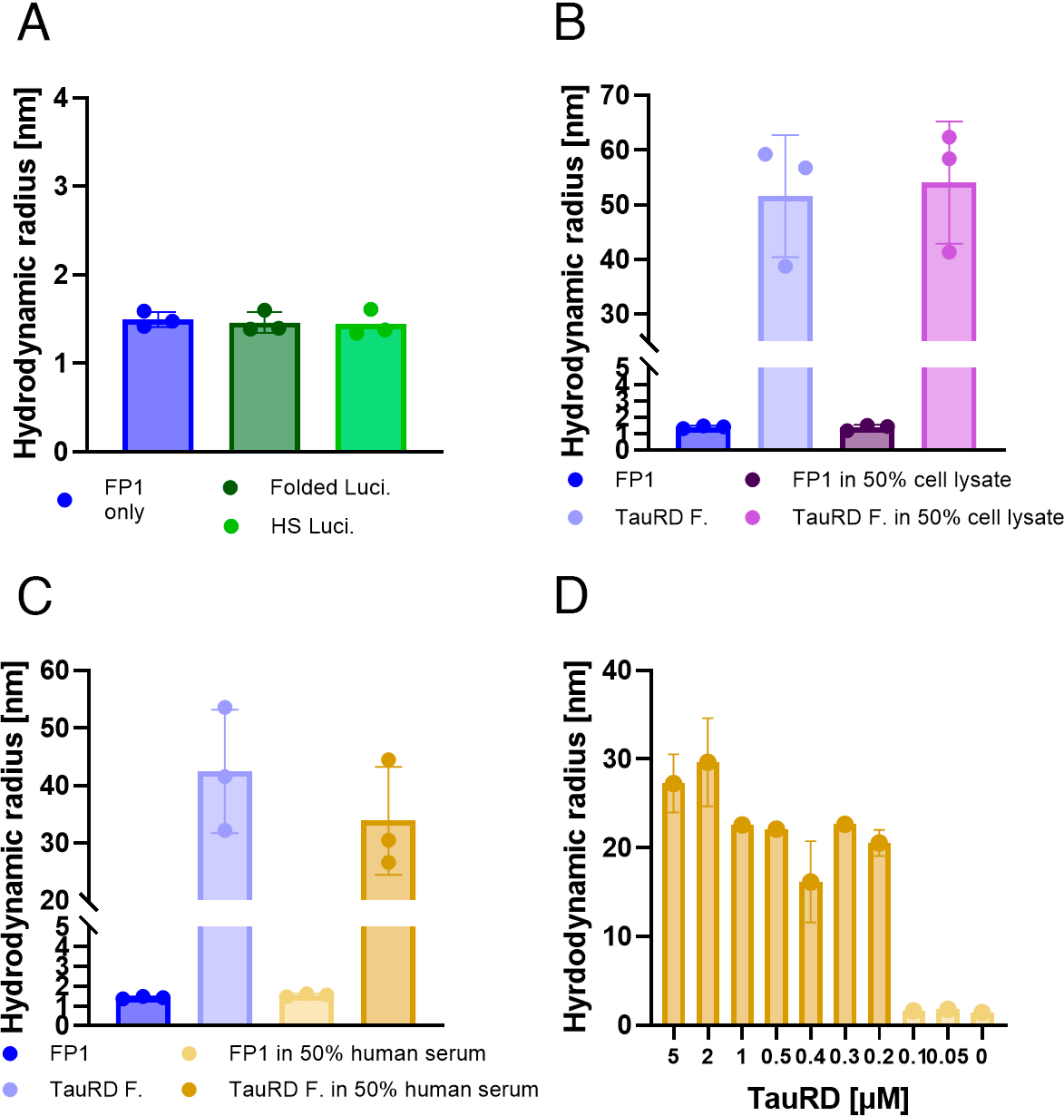
FibrilPaint1 is specific for amyloid aggregates. (*A*) 0.2 μM FibrilPaint1 incubated with amorphous aggregated Luciferase by heat shock. FibrilPaint1 has a R_h_ of 1.7 nm, incubated with heat shock luciferase the size remains the same. (*B*) Binding of 0.2 μM FibrilPaint1 to 2 µM TauRD fibrils in buffer or 50% cell lysate. In buffer, FibrilPaint1 has a R_h_ of 1.7 nm, incubated with TauRD fibrils has an average R_h_ of 52 nm. In 50% cell lysate, FibrilPaint1 has a R_h_ of 1.7 nm, incubated with TauRD fibrils has an average size of 54 nm. (*C*) Binding of 0.2 μM FibrilPaint1 to 2 µM TauRD fibrils in buffer or 50% human serum. In the presence of buffer, FibrilPaint1 has a R_h_ of 1.6 nm, incubated with TauRD fibrils has an average R_h_ of 42 nm. In 50% human serum, FibrilPaint1 has a R_h_ of 1.5 nm, incubated with TauRD, it has an average size of 34 nm. (*D*) Binding of 0.2 μM FibrilPaint1 to different concentrations of TauRD fibrils in 50% human serum. The detection limit of FibrilPaint1 observed under these conditions is 200 nM TauRD fibrils (FibrilPaint1 in buffer, dark blue; folded luciferase, dark green; heat-socked luciferase; light green; TauRD fibrils in buffer, light blue; FibrilPaint1 in cell lysate, dark purple; TauRD fibrils in cell lysate, light purple; FibrilPaint1 in human serum, light yellow; TauRD fibrils in human plasma, dark yellow).

Having confirmed the ability of FibrilPaint1 to bind Tau amyloid fibrils and not monomers, we investigated whether the binding of FibrilPaint1 to aggregates requires amyloid structures. We used Luciferase as an established paradigm for nonamyloid aggregates (45). Luciferase is a globular 61 kDa protein with a R_h_ of 3.4 nm (*SI Appendix*, Table S2) that forms amorphous aggregates when denatured by heat shock. We incubated FibrilPaint1 together with heat-shocked Luciferase in a 1:100 ratio. The R_h_ of FibrilPaint1 in the FIDA measurement is not affected by the presence of luciferase aggregates (Fig. 2*A*). This indicates that FibrilPaint1 does not bind to amorphous luciferase aggregates, demonstrating specificity for amyloid fibrils.

To assess the biochemical specificity of FibrilPaint1, we incubated it with *Escherichia coli* cell lysate, representing a complex mixture of thousands of nontarget proteins. We incubated preformed TauRD fibrils with FibrilPaint1 in a 1:100 ratio in 50% cell lysate. Under these conditions, FibrilPaint1 shows an increased R_h_ of 54 nm, which is consistent with the R_h_ of 52 nm measured from the same fibrils in buffer (Fig. 2*B*). When incubating FibrilPaint1 alone in cell lysate, the R_h_ remains the same as FibrilPaint1 alone (Fig. 2*B*). While *E. coli* lysate is not representative of human biological matrices, this demonstrates the biochemical selectivity of FibrilPaint1 for fibrils in a complex cellular mixture.

To test FibrilPaint1 in a more physiological and disease-relevant environment, we incubated FibrilPaint1 in the absence or presence of a new batch of preformed TauRD fibrils in 50% human serum. FibrilPaint1 alone in 50% human serum appeared to be 1.5 nm, which correlates to the parallel measurement of 1.4 nm observed in buffer (Fig. 2*C*). In presence of preformed TauRD fibrils, the R_h_ increases up to an R_h_ of 42 nm in buffer and 34 nm in 50% human serum (Fig. 2*C*). Differences in the R_h_ are most likely due to fibril heterogeneity. The absence of background signal in serum is necessary for the potential use of FibrilPaint1 for use in clinically relevant environments.

After confirming the ability of FibrilPaint1 to detect TauRD fibrils in human serum, we aimed to estimate the concentration threshold for such measurements. We titrated recombinant TauRD fibrils into serum and measured the R_h_ with the FibrilRuler Test for decreasing concentrations (Fig. 2*D*). TauRD fibrils in this experiment had an average R_h_ value of 21 nm, but this size varies due to the heterogeneity of amyloid fibrils. We can detect these fibrils down to a lower limit of fibrils made from a monomeric solution of 200 nM. With the measured R_h_ value of 21 nm, we can calculate the number of monomers in the average fibril. An R_h_ of 21 nm corresponds to 260 layers for a PHF-shaped fibril (*SI Appendix*, Table S2 and Fig. S5*F*). This means the true fibril concentration is as low as 400 pM. These findings indicate that FibrilPaint1 might be useful to screen for the presence of subnanomolar amyloid concentrations found in pathological settings.

### Monitoring Fibril Kinetics.

We aimed to monitor fibril size throughout the aggregation process by observing the progressive increase in R_h_, using the FibrilRuler Test. To do so, we performed aggregation reactions in the absence of FibrilPaint1. At the desired time, we added FibrilPaint1 to the reaction to measure the R_h_ in FIDA.

**Fig. 3.**
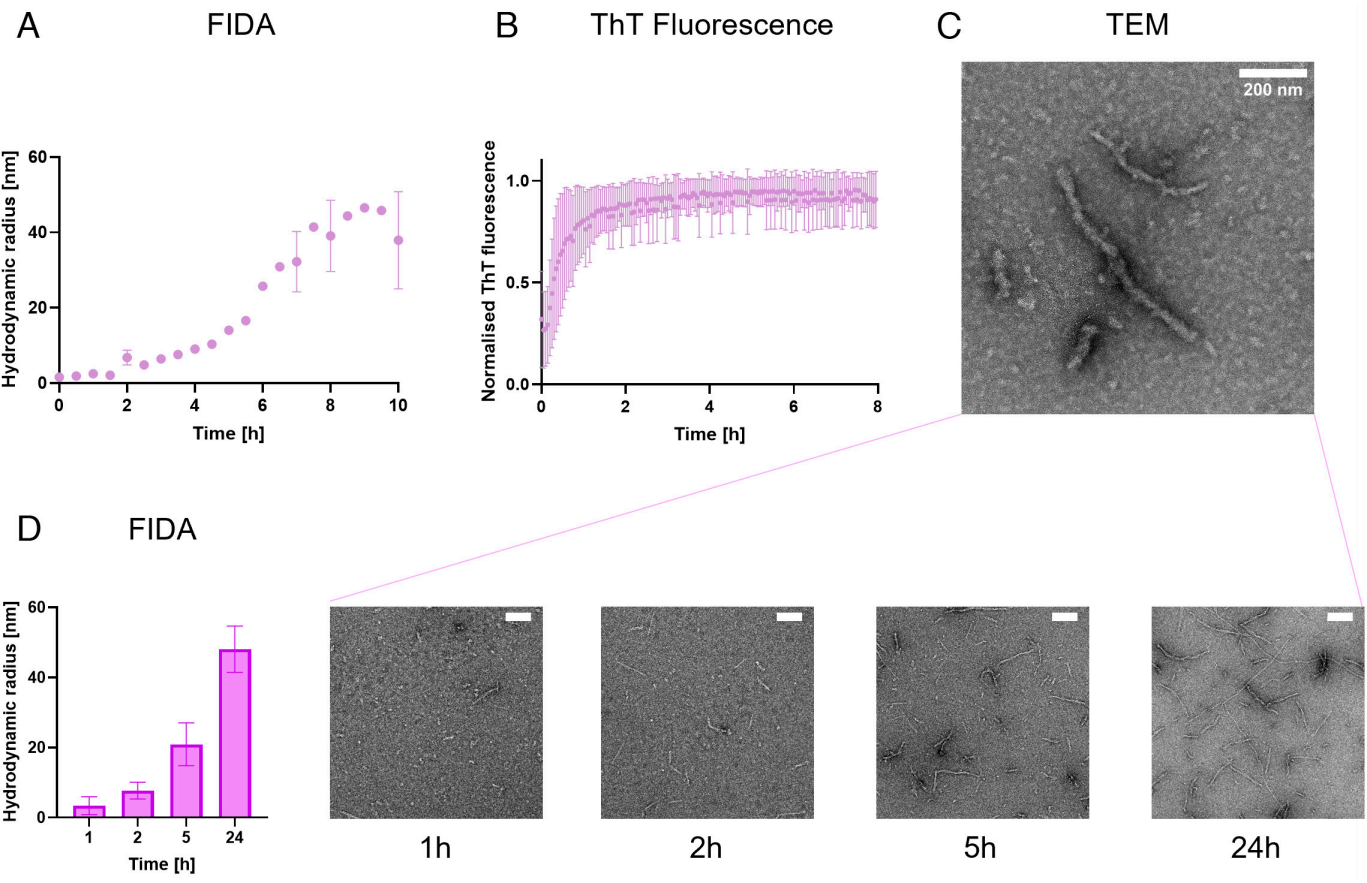
Aggregation of TauRD over time. (*A*) Hydrodynamic radius (R_h_) of TauRD amyloid species made from 20 µM monomers as determined with FIDA. Representative figure shown of N = 3. (*B*) In parallel, aggregation was monitored with a ThT assay. Average of triplicate measurement shown, N = 3. (*C*) Representative transmission electron microscopy (TEM) images of Tau fibrils after 24 h of aggregation. (*D*) Parallel experiments of TauRD aggregation monitored with FIDA (*Left*) and TEM at 1, 2, 5, and 24 h. Average data shown of duplicate measurements. Scale bar in the *Upper Right* corners correspond to 200 nm.

After 0.5 h, the aggregation reaction of TauRD resulted in an increase of the averaged R_h_ from 1.7 nm, which corresponds to FibrilPaint1 alone, to 2 nm (Fig. 3*A*). TauRD aggregation continued and rapidly increased to an R_h_ of 5 nm after 2 h (Fig. 3*A*). TauRD aggregation reached a plateau at an R_h_ value of 45 nm after 8 h.

We monitored the aggregation process in parallel with the established ThT assay (Fig. 3*B*). The ThT fluorescent signal rose immediately after addition of heparin to TauRD and increases rapidly, nearly reaching the plateau phase after 2 h (Fig. 3*B*). After 4 h, no changes were monitored with ThT anymore, but after this point, the TauRD fibrils continue to elongate from an R_h_ of 10 nm to an R_h_ of 45 nm. This suggests continued elongation of the fibrils even after the ThT fluorescence signal reaches saturation. Importantly, different concentrations of the ThT dye did not affect the time the plateau was reached nor the half-time to reach the plateau (*SI Appendix*, Fig. S1). The plateau we observed is therefore independent of the ThT concentration. Continued elongation after this point is in line with previous studies showing the ThT signal does not stringently correlate with amyloid mass (46–48). Possibly, structural maturation could alter ThT binding characteristics over time, decoupling ThT fluorescence from fibril growth. This may reflect that the precise mechanism of ThT–amyloid interaction is not fully understood yet.

To confirm ongoing fibril elongation beyond 2 h, we monitored fibrils in parallel using transmission electron microscopy (TEM). TEM imaging after 24 h of aggregation revealed long, single fibrillar structures (Fig. 3*C*). Monitoring TauRD fibril formation over time by TEM showed that after 1 only a small number of short fibrils were visible, with an average length of 120 nm (Fig. 3*D*). Length but foremost fibril numbers increased at 2 h, to 140 nm, in line with both the ThT signal, and our FibrilRuler Test (Fig. 3 *A*, *B*, and *D* and *SI Appendix*, Fig. S3). However, after 5 h, more and longer fibrils appeared, with an average of 290 nm. Their length continued to increase to 580 nm at 24 h (*SI Appendix*, Fig. S3). Due to manual analysis of the fibril length, a slight bias in favor of larger species may occur. Thus, the FibrilRuler Test provides a direct measurement of fibril size, revealing continued elongation throughout the aggregation range.

To evaluate the sensitivity of the FibrilRuler Test to early-stage fibril formation, we performed repeated aggregation experiments with TauRD and monitored fibril development using FIDA at short time intervals. Experiments always resulted in fibril formation within 150 min, confirming (*SI Appendix*, Fig. S4*A*). Notably, around 100 min, one of the replicates displayed a distinct population with a R_h_ of 4 nm, while other experiments still showed smaller species (2 to 2.4 nm), indicative of differences in startpoints in aggregation. Repeating the analysis for only the first 60 min (*SI Appendix*, Fig. S4*B*) revealed discrete species with R_h_ values of 1.7 nm (corresponding to unbound FP1), 2.0 nm, 2.4 nm, and 3.0 nm.

### Correlating Hydrodynamic Radius to Fibril Length.

**Fig. 4.**
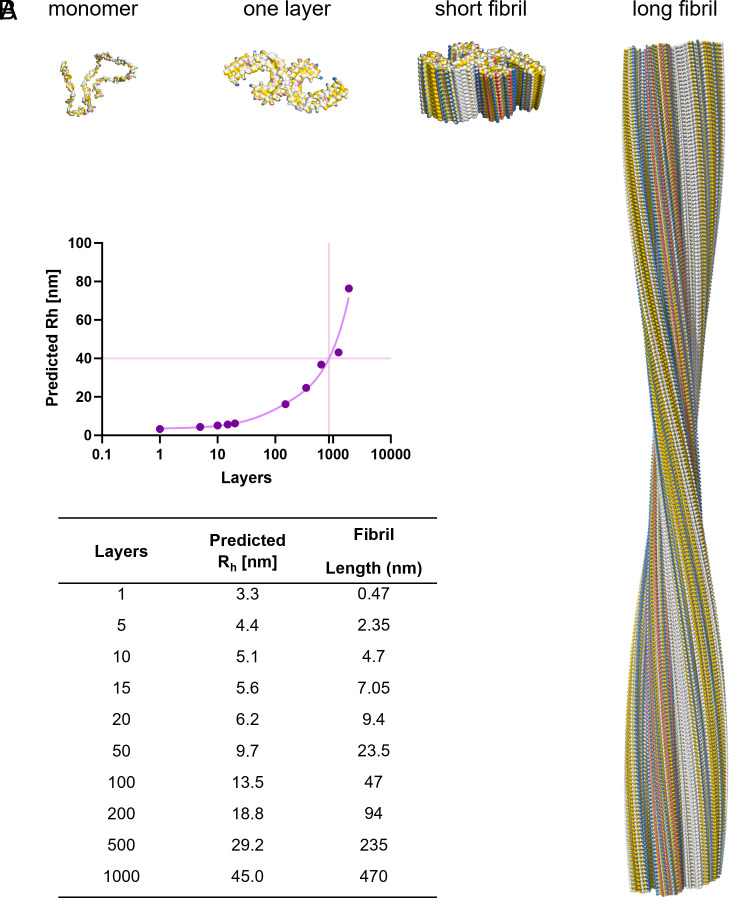
Correlation between fibril length and hydrodynamic radius (R_h_). (*A*) Structures of Tau monomer and Paired Helical Filament fibril as one layer, 20 layers, and 342 layers. (*B*) R_h_ prediction based on the coordinates of the stacked fibril layers. Fibril length is calculated using 0.47 nm for one layer (49).

To correlate the readout of TEM to the FibrilRuler measurements, we built a model to convert the hydrodynamic radius to fibril length. For Tau, many different cryo-EM structures are now available, providing us conformational information (49–52). For TauRD, however, there is no cryo-EM structure available. We chose to use the PHF of AD as a model. The length of the PHF can be determined through its turns, which we used to verify our model. One full turn of the twist of a PHF comprises 342 layers. As the fibril structure is 0.47 nm per layer, a full twist corresponds to a length of approximately 160 nm (49). We stacked in silico layers on top of each other and calculated the predicted with the R_h_ with FIDAbio R_h_ prediction tool (Fig. 4*A*). As a result, we have a mathematical equation to convert the R_h_ to fibril layers and length (Fig. 4*B*).

The length of TauRD fibrils at 24 h varied, 500 to 600 nm on average (Fig. 3*C*). This is slightly longer than we find with the FibrilRuler Test, where we measure a length of roughly 470 nm (Fig. 3*A*). This is expected, as we often exclude smaller fibrils due to the limited visible structure, making it difficult to be certain of the identity of a particle as an amyloid fibril.

The need for measuring fibrils directly in fluid becomes clear at the start of the aggregation process. At 1 h, an average R_h_ of 4 nm was determined. Assuming a PHF fibril structure, this correlates to approximately 4 layers, and a fibril length of 2 nm (Fig. 4*B*). In TEM, we were unable to include fibrils with a fibril length below 80 nm, which is half a turn in the structure, as we were uncertain of the fibril identity. After 2 h, the R_h_ increased 5 nm, corresponding to approximately 10 layers and a fibril length of 4.7 nm (Fig. 4*B*). It is, however, likely that early fibrils take on other conformations with smaller interfaces (28, 29). Thus, the FibrilRuler Test allowed detecting early species involved in aggregation, offering the possibility to screen for Tau amyloid fibrils early in the disease progression.

### FibrilPaint1 Detects Patient-Derived Fibrils.

**Fig. 5.**
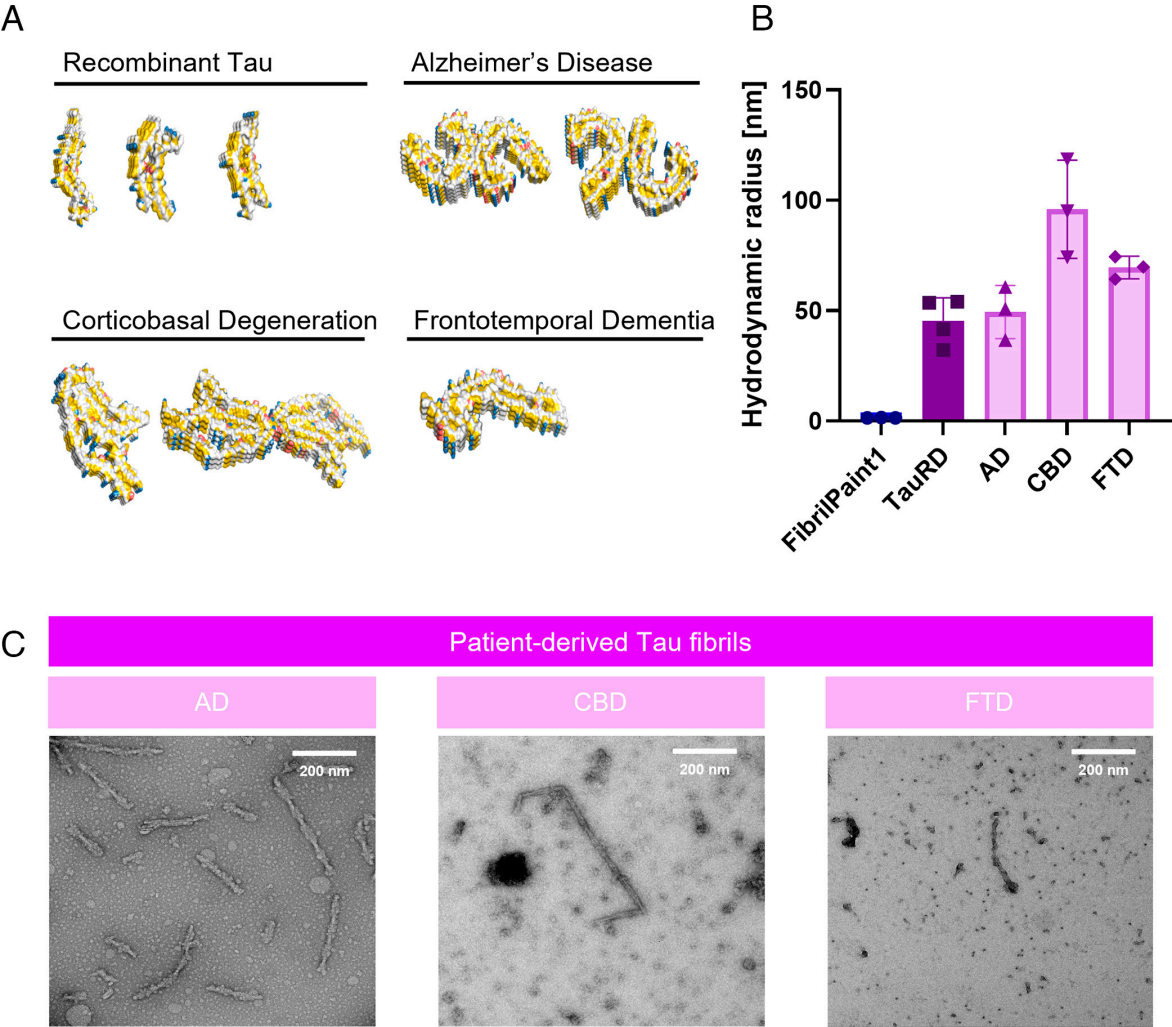
Size assessment of patient-derived material from various tauopathies. (*A*) Structural representation of the Tau fibrils of different tauopathies and recombinant Tau fibrils for comparison. The images were generated in PyMol software based on the coordinates of the cryo-EM structures. Functional groups are colored according the YRB script. PDB codes: AD paired helical filament, 5O3L; AD straight filament, 5O3T; FTD narrow filament, 6GX5; CBD type I 6TJO; CBD type II 6VH7; Snake recombinant Tau, 6QJH; twister recombinant Tau, 6QJM; Jagged recombinant Tau 6QJP [Colors in YRB: yellow; negative charge, red; positive charge, blue (53)]. (*B*) Hydrodynamic radius of fibrils extracted from patients diagnosed with Alzheimer’s diseases (AD), frontotemporal dementia (FTD), and corticobasal degeneration (CBD), measured with FibrilPaint1. (FibrilPaint1 only, blue; TauRD fibrils, dark purple; fibrils retrieved from patients, light purple). (*C*) Representative TEM images of patient-derived Tau fibrils from AD, CBD, and FTD (FLTR). White Scale bar in the *Upper Right* corners correspond to 200 nm.

As a potential reporter for Tau fibrils, it is important to assess the ability of FibrilPaint1 to recognize patient-derived fibrils of several Tauopathies. Interestingly, cryo-EM structures of Tau fibrils show that their shape differs for the various tauopathies and heparin-induced recombinant fibrils (10) (Fig. 5*A*). Therefore, we set out to measure ex vivo Tau fibrils from three different Tauopathies. We purified fibrils from deceased patients diagnosed with CBD, FTD, and AD (49, 50, 52). Monomeric Tau undergoes different posttranslational alternative splicing in different diseases, leading to six different isoforms, with either four (4R) or three (3R) repeats of the microtubule-binding domain. Depending on the isoform incorporated in the fibril, tauopathies can be classified as 4R (CBD), 3R (FTD), or 4R/3R (AD). These different isoforms also have different conformations in each disease (10).

Using our microfluidics setup, we could detect all three tauopathies (Fig. 5*B*). The fibrils formed by recombinant TauRD had an average R_h_ of 45 nm. Fibrils measured from a patient diagnosed with AD had an average R_h_ of 49 nm, with little variation between the measurements (Fig. 5*B*). For CBD, the observed R_h_ was 95 nm, with more variation in between measurements, likely reflecting a heterogeneous population of fibrils in the patient’s brain (Fig. 5*B*). Fibrils from FTD appeared to be a homogeneous population, with an average R_h_ of 69 nm (Fig. 5*B*).

In the case of AD, the average R_h_ of 49 nm corresponds to a length of 510 nm or 1,100 layers, as given by our FIDAbio prediction tool (Fig. 4*B*). We imaged the purified fibrils with TEM (Fig. 5*C*). For AD, we manually investigated over 100 fibrils, of which half showed the typical PHF structure and an average 2.7 turns (*SI Appendix*, Fig. S5 *C* and *D*). With the known repetitive element in these fibrils, this corresponds to 430 nm. Of course, this excludes the Straight Filaments found in the same fibril population. The order of magnitude is in line with the 510 nm observed in the FibrilRuler Test.

We compared these results to the TauRD fibrils, which had a similar R_h_ of 45 nm. We measured fibril length directly, without the use of any structural elements. Quantification was restricted to fibrils entirely visible in the defined analysis area, including a total of over 100 species (*SI Appendix*, Fig. S5 *C* and *D*). The average fibril length was 550 nm. Our findings demonstrate that the FibrilRuler Test is an effective tool for assessing the length of pathological fibrils. However, fibril length alone does not allow for differentiation between fibril types. Therefore, complementary techniques such as TEM or fibril-specific antibodies are required to accurately determine fibril identity.

### FibrilPaint1 Binds Other Amyloid Structures.

**Fig. 6.**
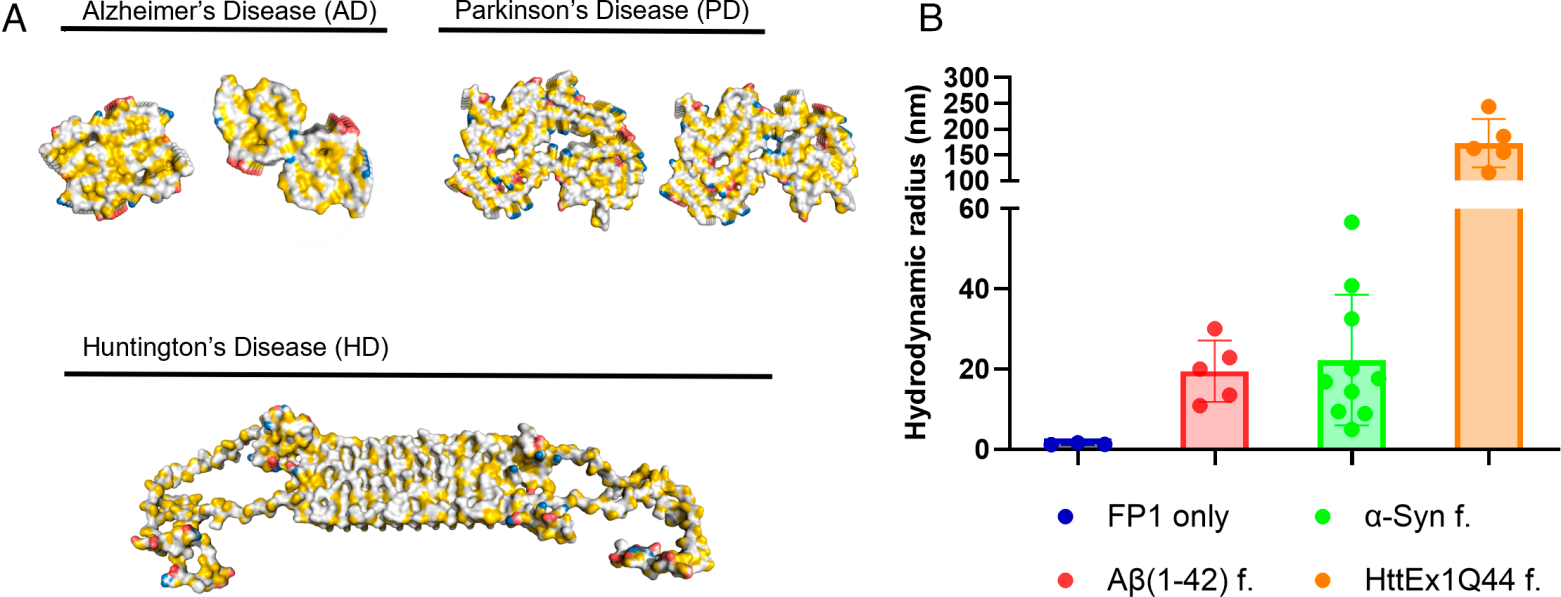
FP1 binds other amyloids. (*A*) *Top* view of cryo-EM structures of fibrils formed by Aβ42 (60) and α-Syn (61) and NMR structure of HttEx1Q44(58), colored in YRB [Colors in YRB: yellow; negative charge, red; positive charge, blue (53)]. (*B*) Hydrodynamic radius of FibrilPaint binding (only, blue) to fibrils formed by Aβ42 (red) α-Syn (green) and HttEx1Q44 (orange).

Finally, we tested whether FibrilPaint1’s selectivity for Tau fibrils over monomers through π-stacking interactions by testing FibrilPaint1’s binding to other amyloid fibrils. Although distinct proteins are involved, amyloid fibrils share a comparable fold, which may lead to a common binder. We selected three proteins known to form other amyloid fibrils; Aβ, α-Synuclein (α-Syn), and Huntingin Exon 1 (HttEx1). The deposition of the Aβ peptide–forming plaques is linked to the development of AD (54, 55). α-Syn fibrils are associated with synucleinopathies like Parkinson’s Disease (56, 57). HttEx1 comprises a polyglutamine stretch of which the length correlates to the onset and severity of Huntington’s Disease (HD), an autosomal inherited disorder (58, 59). These amyloid fibrils are unrelated in sequence and structure but have β-sheet formation in common (Fig. 6*A*).

Remarkably, FibrilPaint1 binds to all three amyloids. Binding to Aβ42 fibrils revealed an average R_h_ of 19 nm. The α-Syn fibrils showed an Rh of 20 nm. Fibrils formed by HttEx1Q44 revealed the largest size, with an average Rh of 160 nm (Fig. 6*B*). This may be related to the very fast growth of poly-Q fibrils after cleaving the MBP fusion protein, which is a known phenomenon. Thus, these data show that FibrilPaint1’s specificity is not limited to Tau amyloid fibrils but extends to other amyloid structures as well.

## Discussion

We have developed FibrilPaint1, a peptide capable of recognizing Tau amyloid fibrils in solution with high affinity, both recombinant and patient-derived fibrils from three distinct tauopathies. This provides a molecular ruler for Tau fibrils, measuring fibril length from 4 to 1,100 layers. FibrilPaint1 is applicable in complex environments such as human serum, where we can detect fibrils in subnanomolar concentrations. FibrilPaint1 is, therefore, a tool to assess the length of Tau amyloid fibrils throughout the aggregation process.

Quantifying amyloid fibril length in solution provides a structurally informative parameter that complements existing techniques. This parameter helps to assess fibrils and monitor their growth or shrinkage. It is known that smaller fibrils may interact more with the cellular environment and disrupt membranes more easily than longer fibrils (62–64). Disaggregation may cause the disease to accelerate when the resulting species are still seeding competent (65). This information goes beyond ThT or antibody-based detection assays, which cannot distinguish small from large aggregates (30, 31). Size information in solution is complementary to imaging data obtained by microscopy techniques, which are capable of distinguishing fibril morphologies but lack scalability for widespread application (66–68).

The noncovalent, fluorescent-labeling of amyloid fibrils with FibrilPaint1 allows studying these fibrils in complex samples. While the binding mechanisms of widely used amyloid dyes such as Thioflavin T and Congo Red are not yet fully resolved, they are thought to intercalate within the β-sheet structure of fibrils (30, 31, 33, 69). Small molecules such as Flortaucipir and EGCG form repetitive stacks framed by the fibril fold (19, 70). Tau fibrils aberrantly attract proteins enriched in π-stacking residues, a category to which FibrilPaint1 may also belong (35). The repetitive nature of fibrils increases the importance of such low-affinity contacts, creating the avidity effect, and potentially introduces a bias favoring longer fibrils over shorter ones (34, 35). Uncovering the molecular binding mechanism of FibrilPaint1 is therefore critical to understand its performance and limitations in complex samples and for the rational design of next-generation fibril-targeting probes.

The FibrilRuler Test cannot differentiate the nature of amyloid fibrils. For biochemical studies, this does not pose a problem, as the nature of the fibril is already known. For diagnostics purposes, this would require independent identification of the nature of the fibril, such as using specific antibodies as complementary tool. The specificity of FibrilPaint1 for amyloid fibrils could also be interesting for developing novel warheads for Targeted Protein Degradation (TPD). TPD is based on bifunctional degraders, of which one side binds specifically to the target, the warhead, and the other targets the protein degradation machinery (71–74). Mixed pathologies are common in neurodegenerative diseases (75–77). A compound capable of targeting this diversity could offer a significant therapeutic advantage by addressing the full spectrum of pathological features within a single treatment approach.

Seed amplification assays have demonstrated that seeding competent species are present in CSF and blood serum prior to the onset of clinical symptoms (78–83). Notably, aggregates found in the CSF seem to elongate with AD progression (66, 67). Future efforts with the FibrilRuler Test could be to study these fibrils directly in patient samples. This offers the interesting possibility to screen patient samples for indication of neurodegeneration or study the correlation of fibril length to disease stage.

## Materials and Methods

A detailed description of all methods is provided in *SI Appendix*.

### TauRD Expression.

FLAG-tagged TauRD (ΔK280) was produced in *E. coli* BL21 Rosetta 2 (IPTG, 18 °C, overnight), captured on Ni-NTA via an N-terminal His_6_-Smt tag, released with Ulp1, polished by cation-exchange and Superdex 75, concentrated, and stored at –80 °C.

### Firefly Luciferase.

His_6_-luciferase was expressed in XL10-Gold cells (IPTG, 20 °C) and purified by Ni-NTA; A_280_ provided concentration, SDS-PAGE purity, and aliquots were frozen at –80 °C.

### Peptide Synthesis.

Twenty-two-residue “FibrilPaint” peptides were made by Fmoc SPPS, N-terminally carboxy-fluorescein-labeled, TFA-cleaved, ether-precipitated, RP-HPLC-purified, and verified by ESI-MS/analytical HPLC.

### TauRD Aggregation Assay.

Twenty µM TauRD + 5 µM heparin were incubated ± 45 µM ThT in 25 mM HEPES-KOH pH 7.4, 75 mM KCl/NaCl (37 °C). ThT fluorescence (440/485 nm) was recorded every 5 min for 24 h on a CLARIOstar® Plus; peptides were added as indicated.

### Aβ42, α-Synuclein, HttEx1Q44 Fibrils.

Aβ42 in PBS was frozen (–20 °C), thawed, and aged 20 h at 37 °C + NaN_3_; α-synuclein (50 µM) was shaken 17 d at 37 °C; HttEx1Q44 (20 µM) was released from an MBP-His_6_ fusion with 0.5 µM Factor Xa and fibrillized 4 h at 37 °C. Aggregation was confirmed by AFM or pelleting.

### Patient-Derived Tau Fibrils.

Sarkosyl-insoluble fractions from AD, FTD, and CBD cortex were isolated by differential centrifugation/detergent washes (CBD extractions included amphipol). Pellets were stored at 4 °C and checked by TEM.

### Electron Microscopy.

Samples were applied to glow-discharged carbon grids, stained with 2 % uranyl acetate, and imaged on a Talos L120C (120 kV, ≤120 k ×). Fibril lengths were measured in Fiji; AD PHF lengths were inferred from helical pitch.

### FibrilRuler (FIDA).

Preformed or patient-derived fibrils (2 µM monomer) were mixed with 50 nM Fl-FibrilPaint1 and analyzed in capillary dissociation mode (25 °C, FIDA 1). Hydrodynamic radii (R _ h) of the largest population were converted to length using a cylindrical model (*SI Appendix*, Eq. 1).

### Microscale Thermophoresis.

FibrilPaint1 (50 nM) was titrated with TauRD monomer or fibrils (0.5 nM to 10 µM) at 37 °C (Monolith NT.115); binding was extracted from the 5 s MST response.

### R_h_-To-Length Conversion.

A PyMOL model of stacked PHF layers (0.47 nm per layer) provided a calibration curve for L/R > 5, enabling direct translation of R_h_ into mean fibril length.

## Supplementary Material

Appendix 01 (PDF)

## Data Availability

All study data are included in the article and/or *SI Appendix*.
