# Supplementary material for: FibrilPaint to determine the length of Tau amyloids in fluids
Source: Proc Natl Acad Sci U S A. 2025 Oct 27;122(44):e2502847122. doi: 10.1073/pnas.2502847122 (PMC12595476; doi:10.1073/pnas.2502847122)
Supplement: Supplementary file 1 — Appendix 01 (PDF) [file pnas.2502847122.sapp.pdf]

PNAS

Supporting Information for

FibrilPaint to determine the length of Tau amyloids in fluids

Júlia Aragonès Pedrola[1, 2, 3, #], Françoise A. Dekker[1, 2, 3, #], Tommaso Garfagnini[4, 5] Guy Mayer[4], Margreet B. Koopman[1, 2, 3], Menno Bergmeijer[3, 6], Gobert Heesink[7], Iris Rots[1, 2], Mireille M. A. E. Claessens[7], Friedrich Förster[3, 6], Jeroen J. M. Hoozemans[8], Henrik Jensen[9], Assaf Friedler[4, *], Stefan G. D. Rüdiger[1, 2, 3 *]

*Corresponding authors

Stefan G. D. Rüdiger; s.g.d.rudiger@uu.nl
Assaf Friedler; assaf.friedler@mail.huji.ac.il

This PDF file includes:

Suppl. Figures S1 to S5
Table S1 to S3
Extended Materials & Methods section

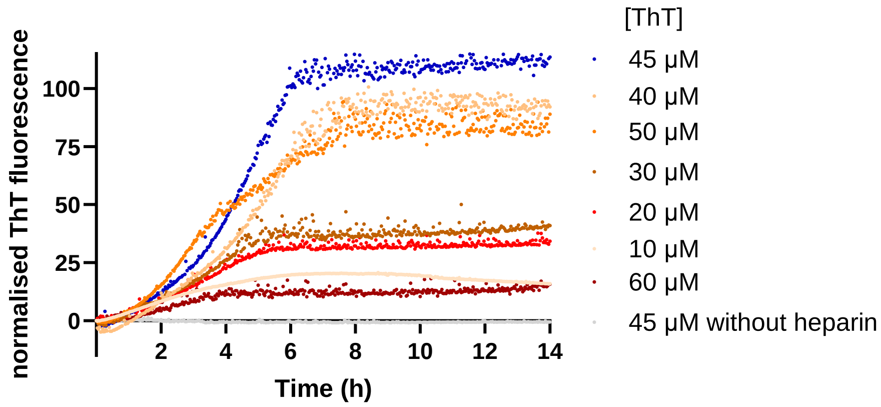

Suppl. Figure S1. **At 45 μM ThT delivers the largest window to study the kinetics of 20 μM TauRD aggregation**. Aggregation was initiated by adding 5 μM heparin to 20 μM TauRD. Reactions were monitored at 10, 20, 30, 40, 45, 50, and 60 μM ThT; all traces are normalised to the maximal signal obtained (at 45 μM, blue) for direct comparison. The half-time of conversion (t_{50}) is 4.2 ± 0.4 h and the fluorescence plateau is reached at 6 h. For the t_{50} calculation, data at 10 μM and 60 μM ThT were omitted because their limited signal window made kinetic fits unreliable. Data shown are the mean n = 3 independent experiments, each run in duplicate technical replicates. The legend on the right lists the traces in order of their peak signal amplitude.

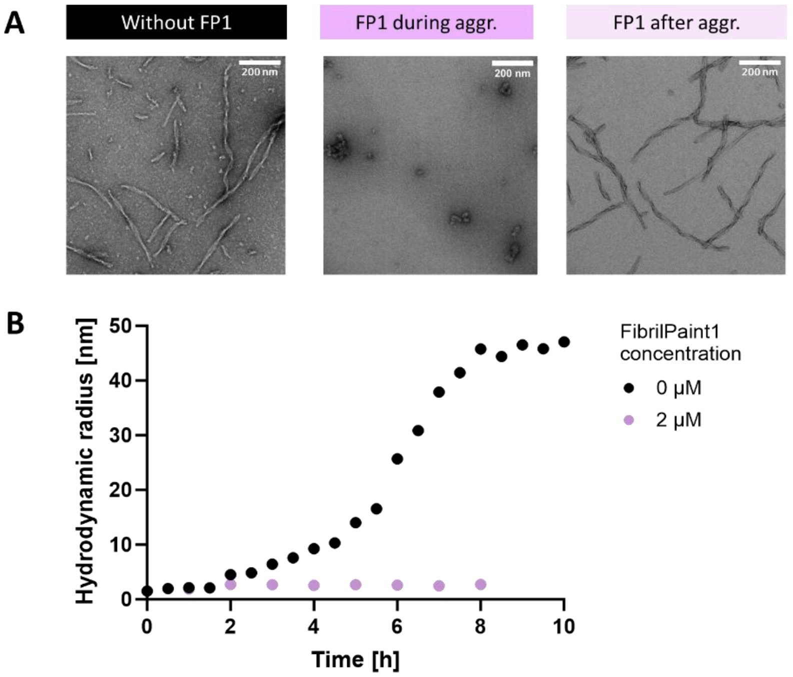

Suppl. Figure S2. **FibrilPaint1 inhibits TauRD fibril formation. A)** TauRD fibril structures made from 20 µM monomers without the presence of FibrilPaint1 (FP1) (left), with 2 µM of FP1 present during aggregation (middle), or with 2 µM of FP1 added after aggregation (right). **B)** **I**nhibited Tau species by FibrilPaint present during aggregation, measured by FIDA (purple), showing species with an Rh of 3.5 nm to 4 nm, compared to aggregation curve of Tau measured when FibrilPaint1 is added after aggregation (Fig 3A, black).

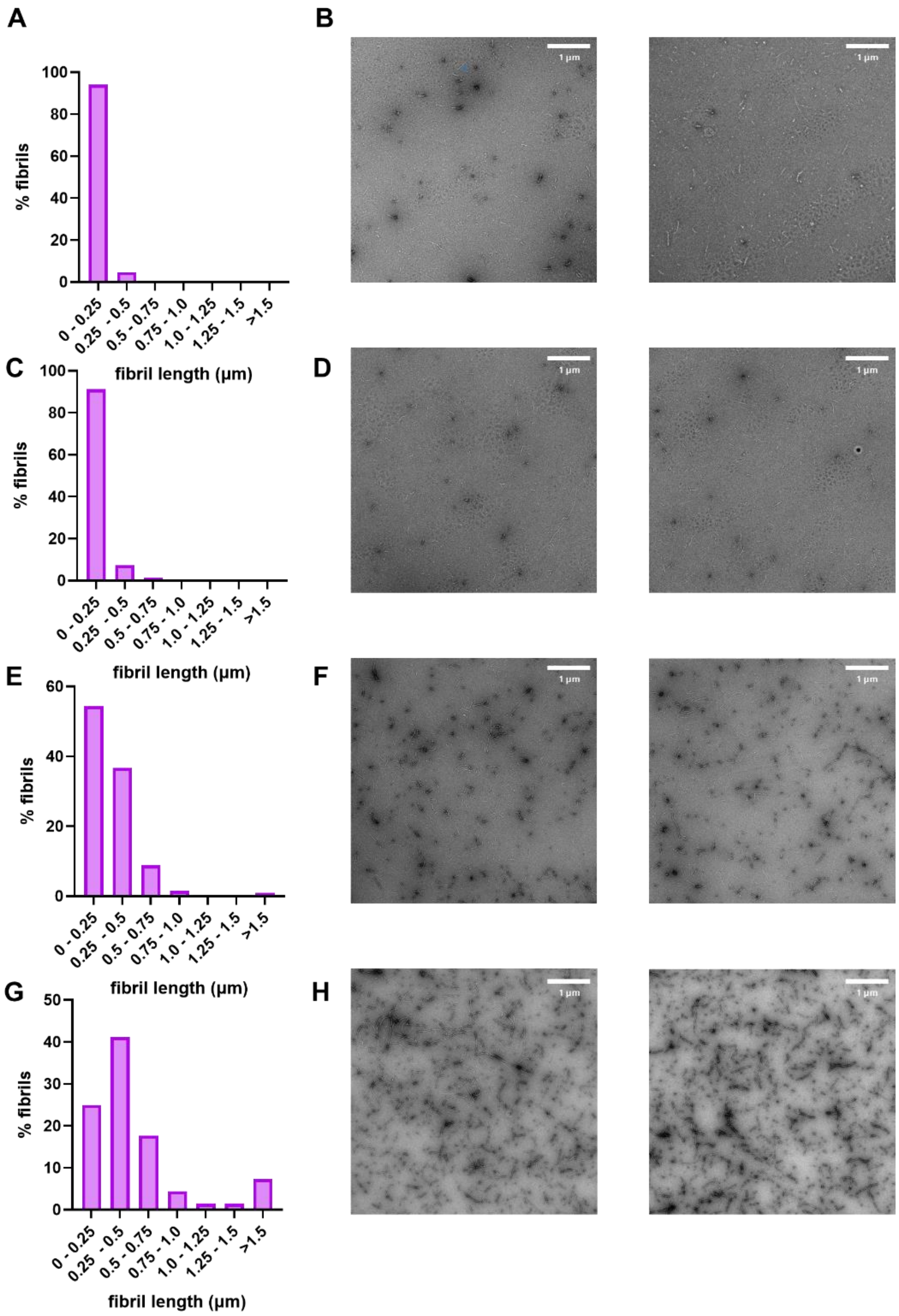

Suppl. Figure S3. **Fibril length analysis of electron microscopy images during TauRD aggregation at different time points.** Samples were collected and analyzed after (A, B) 1 hour, (C, D) 2 hours, (E, F) 5 hours, and (G, H) 24 hours of aggregation. Histograms in panels A, C, E, and G display the distribution of fibril lengths as a percentage of the total fibril population. Panels B, D, F, and H show two representative microscopy fields per time point with annotated fibrils for visualization. Scale bar in the upper right corner of each image represents 1 µm.

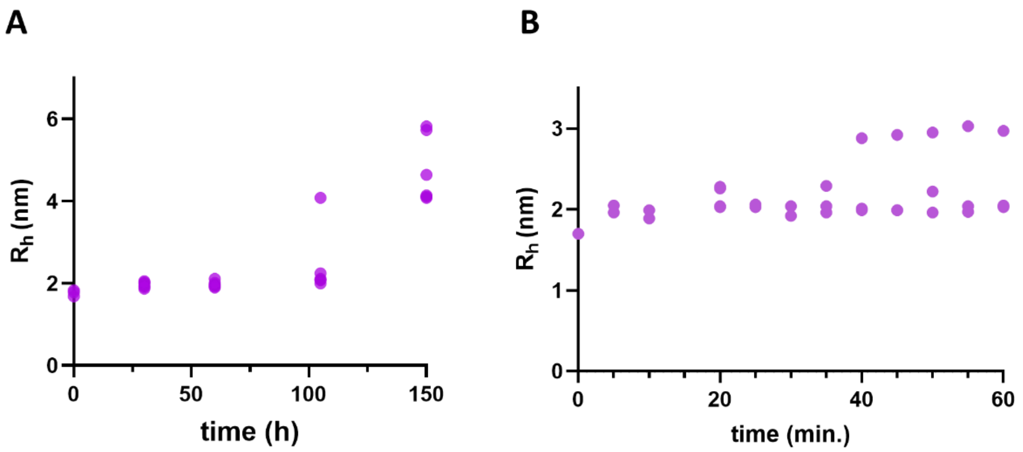

Suppl. Figure S4. **FIDA measurements during early aggregation of 20 µM TauRD. (A)** Results from four independent experiments, measured up to 150 minutes. After 150 minutes, all experiments consistently show clear aggregation. A notable shift occurs around 100 minutes, where one experiment detects a species with a hydrodynamic radius (R_h) of 4 nm, while the others remain between 2–2.4 nm. **(B)** Repetition of early aggregation timepoints (0–60 minutes) for three experiments. Distinct R_h species are observed: 1.7 nm (corresponding to unbound FP1), 2.0 nm, 2.4 nm, and 3.0 nm. Each experiment shows aggregation onset at slightly different time points.

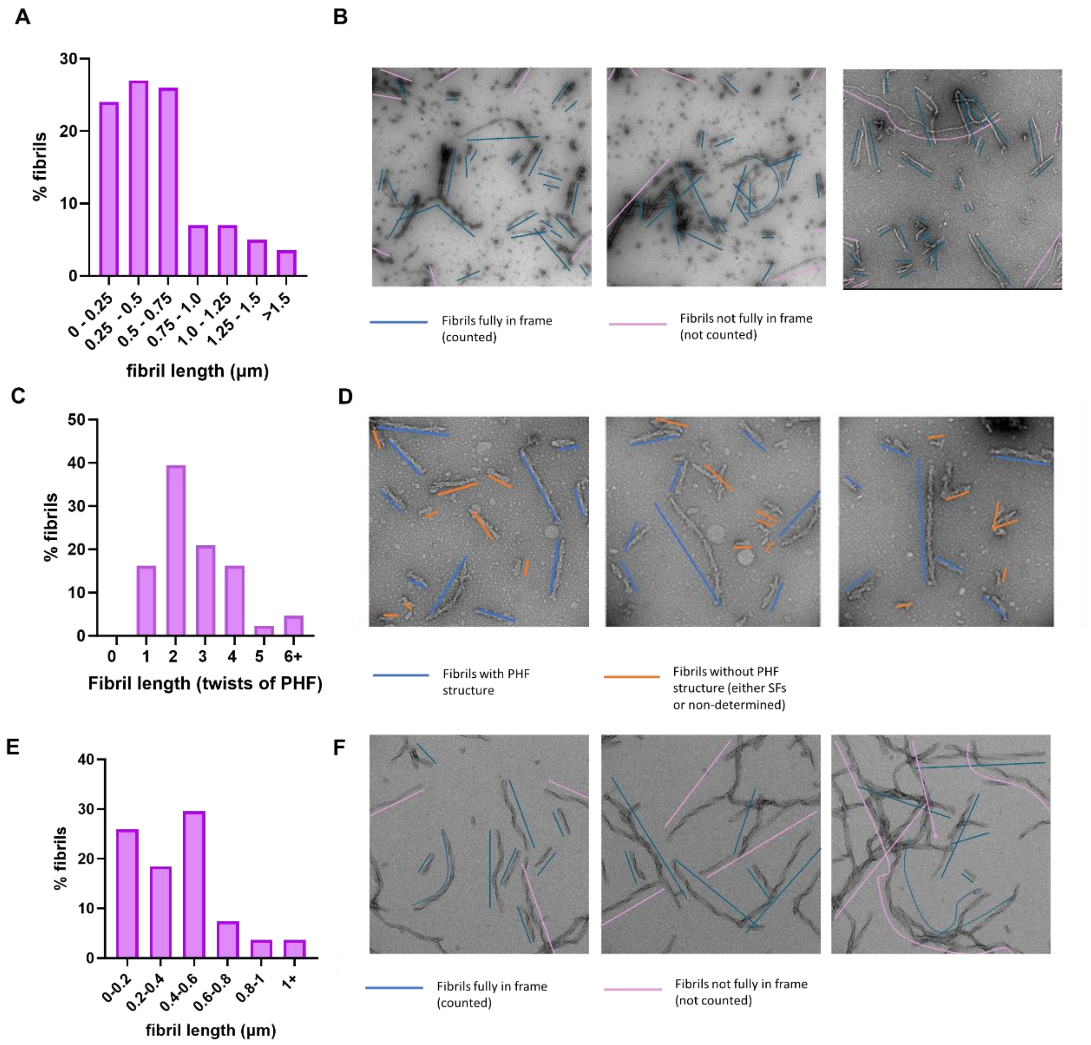

Suppl. Figure S5. **Overview of fibril length analysis for TauRD and Alzheimer-derived fibrils. (A, B)** Fibrils formed by 20 µM TauRD after 8 hours in vitro aggregation. **(C, D)** Fibrils extracted from Alzheimer's Disease (AD) brain tissue, estimated concentration 2 µM. **(E, F)** Fibrils grown by 20 µM TauRD in vitro and treated with FP1 after 8 hours. Histograms (A, C, E) represent the average fibril length, based on at least 100 manually measured fibrils, expressed in µm (A,E) or in twists of PHF fibrils (C). Three annotated example images per group (B, D, F) illustrate how fibrils were selected and measured, with blue fibrils indicating fibrils included, while pink indicate out of square or orange non-twisting fibrils.

Suppl. Table S1. **Peptide names and sequences.** (Fl) at the N-terminal of the sequence indicates fluorescein, which is not considered when calculating the molecular weight or number of amino acids. Amino acid colouring is based on YRB colours (hydrophobic residues, yellow; negatively charged residues, red; positively charged residues, blue) (1) Net charge is the total charge of the different peptides at physiological pH. Fibril binding is the detection of Tau amyloid fibrils with Flow Induced Dispersion Analysis (FIDA). Hydrodynamic Radius (R_h) is theradius determined with FIDA when the peptides do not bind anything.

**Name peptide**	**Sequence**	**Net charge**	**MW (kDa)**	**Tht inhi-bition**	**Fibril binding**	**R_h (nm)**
aB Peptide*	PWIRRPFFPFHSPSR	+3	1.93	Yes	-	
WTG peptide*	GWWRRGWWGWTTGTR	+3	1.95	Yes	-	
FibrilPaint1	(Fl)-PWWRRPWWPWHHPHGSGSEEVD	-1	2.82	Yes	Yes	1.7
FibrilPaint2	(Fl)-PWIRRPWWPWTTPTGSGSEEVD	-1	2.64	Yes	No	1.7
FibrilPaint3	(Fl)-PWWDDPWWPWTTPTGSGSEEVD	-5	2.63	Yes	No	1.7
FibrilPaint4	(Fl)-PWWDDPWWPWHHPHGSGSEEVD	-5	2.74	Yes	No	1.7

Suppl. Table S2. **Protein structures and their predicted hydrodynamic radii**. The number of residues and the molecular weight are given for the total structure as available in the PDB. If structures are available, the included 1 layer R_h is the R_h a structure would have if it could fold in its fibrillar conformation with just one layer. The 5 layers R_h is the R_h that results from a fibril that stacks 5 layers. Origin of the structure is the method used to determine the coordinates of PDB structures.

Protein structure	PDB code	Nr. residues	MW (kDa)	Predicted R_h (nm) 1 layer	5 layers	Origin structure
Snake recombinant Tau	6QJH	177	18.67	2.29	3.49	Cryo-EM (2)
Twister recombinant Tau	6QJM	144	15.57	1.73	2.68	Cryo-EM (2)
Jagged recombinant Tau	6QJP	144	15.57	1.75	2.70	Cryo-EM (2)
TauRD monomer		128	14.21	4.16		AlphaFold
Tau AD Paired helical filament	5O3L	730	79.40	3.33	4.33	Cryo-EM (3)
Tau AD straight filament	5O3T	730	79.40	3.29	4.34	Cryo-EM (3)
FTD narrow filament	6GX5	282	30.41	2.84	4.32	Cryo-EM (4)
CBD type I	6TJO	321	137.76	2.67	4.07	Cryo-EM (5)
CBD type II	6VH7	642	69.65	4.12	6.25	Cryo-EM (5)
Luciferase	1LCI	550	60.82	3.35		X-Ray (6)
Recombinant alpha-synuclein fibril	6CU7	140	144.76	2.66	3.53	Cryo-EM (7, 8)
Recombinant amyloid-β(1 -42) fibril	5OQV	42	40.68	2.41	3.15	Cryo-EM (9)
HttEx1Q44 fibril				5.32	7.28	NMR (10)

Suppl. Table S3. **Experimental set-up used in FIDA Experiments.** Tray 1 was maintained at 37°C and tray 2 and capillary chamber at 25°C.

Tray	Vial	Pressure (mbar)	Time (s)	Outlet	Measure	Comments
2	1	3500	45	Variable	No	1 M NaOH
2	2	3500	75	Variable	No	Buffer equilibration
1	Analyte	3500	20	Variable	No	Buffer
1	Indicator	50	10	Variable	No	Sample application
1	Analyte	75	1800	Variable	Yes	Buffer

Extended Materials and Methods

Expression and purification of TauRD

We produced N-terminally FLAG-tagged (DYKDDDDK) human TauRD (Q244-E372, with pro-aggregation mutation ΔK280) in *E. Coli* BL21 Rosetta 2 (Novagen), with an additional removable N-terminal His_6-Smt-tag (MGHHHHHHGSDSEVNQEAKPEVKPEVKPETHINLKVSDGSSEIFFKIKKTTPL RRLMEAFAKRQGKEMDSLRFLYDGIRIQADQTPEDLDMEDNDIIEAHREQIGG). Expression was induced at OD_{600} 0.8 by addition of 0.15 mM IPTG and incubation at 18 ^{o}C overnight. Cells were harvested by centrifugation, resuspended in 25 mM HEPES-KOH pH=8.5, 50 mM KCl, flash frozen in liquid nitrogen, and kept at – 80 ^{o}C until further usage. Pellets were thawed at 37 ^{o}C, followed by the addition of ½ tablet/50 ml EDTA-free Protease Inhibitor and 5 mM β-mercaptoethanol. Cells were disrupted using an EmulsiFlex-C5 cell disruptor, and lysate was cleared by centrifugation. Supernatant was filtered using a 0.22 μm polypropylene filtered and purified with an ÄKTA purifier chromatography System. Sample was loaded into a POROS 20MC affinity purification column with 50 mM HEPES-KOH pH 8.5, 50 mM KCl, eluted with a linear gradient 0-100%, 5 CV of 0.5 M imidazole. Fractions of interest were collected, concentrated to 2.5 ml using a buffer concentration column (vivaspin, MWCO 10 kDa), and desalted using PD-10 desalting column to HEPES pH 8.5, ½ tablet/50 ml Complete protease inhibitor, 5 mM β-mercaptoethanol. The His_6-Smt-tag was removed by treating the sample with Ulp1, 4 ^{o}C, shaking, overnight. The next day, sample was loaded into POROS 20HS column with HEPES pH 8.5, eluted with 0-100% linear gradient, 12 CV of 1M KCl. Fractions of interest were collected and loaded into a Superdex 26/60, 200 pg size exclusion column with 25 mM HEPES-KOH pH 7.5, 75 mM NaCl, 75 mM KCl. Fractions of interest were concentrated using a concentrator (vivaspin, MWCO 5 kDa) to desired concentration. Protein concentration was measured using a NanoDrop™ One^{C} UV/Vis spectrophotometer and purity was assessed by SDS-PAGE. Protein was aliquoted and stored at – 80 ^{o}C.

Expression and purification of *Photinus pyralis* Firefly luciferase

We produced His_6-tag firefly luciferase was expressed in XL10 Gold strain (Stratagene, US). After growing cells at 37 ^{o}C, Expression was induced when OD_{600} 0.5, the temperature was lowered to 20 ^{o}C. After 45 min incubating, expression was induced by adding 0.10 M IPTG and incubated overnight. Cells were harvested by centrifugation, and pellets were resuspended in lysis buffer (50 mM $Na_xH_yPO_4$ pH 8.0, 300 mM NaCl, 10 mM β-mercaptoethanol, protease inhibitors (cOmplete, EDTA free, Roche) DNase10 mg/ml). Cells were disrupted using an EmulsiFlex-C5 cell disruptor and lysate was cleared by centrifugation. Super natant was filtered using a 0.22 μm polypropylene filtered and purified with an ÄKTA purifier chromatography System. Sample was loaded onto a POROS 20MC affinity purification column with lysis buffer, and eluted with a linear gradient 0-100%, 5 CV of 0.5 M imidazole. Fractions of interest were collected and dialyzed overnight using dialysis buffer (50 mM $Na_xH_yPO_4$ pH 8.0, 300 mM NaCl and 10 mM β-mercaptoethanol, 10% glycerol). Protein concentration was measured using a NanoDrop™ One^{C} UV/Vis spectrophotometer and purity was assessed by SDS-PAGE. Protein was aliquoted and stored at -80 ^{o}C.

Peptide synthesis and purification

The peptides were synthesized using a Liberty Blue Microwave-Assisted Peptide Synthesizer (CEM) with standard Fmoc chemistry and Oxyma/DIC as coupling reagents. The peptide concentrations were measured by UV spectroscopy. The peptides were labeled with 5(6)-carboxyfluorescein at their N' termini. The peptides were cleaved from the resin with a mixture of 95% (v/v) trifluoroacetic acid (TFA), 2.5% (v/v) triisopropylsilane (TIS), 2.5% (v/v) triple distilled water (TDW) agitating vigorously for 3 hours at room temperature. The volume was decreased by N_2 flux and the peptides precipitated by addition of 4 volumes of diethylether at -20 °C. The peptides were sedimented at -20 °C for 30 minutes, then centrifuged and the diethylether discarded. The peptides were washed three times with diethylether and dried by gentle N_2 flux. The solid was dissolved in 1:2 volume ratio of acetonitrile (ACN):TDW, frozen in liquid Nitrogen and lyophilized. The peptides were purified on a WATERS HPLC using a reverse-phase C18 preparative column with a gradient of ACN/TDW. The identity and purity of the peptides was verified by ESI mass spectrometry and Merck Hitachi analytical HPLC using a reverse-phase C8 analytical column.

TauRD aggregation

Aggregation of 20 µM TauRD in 25 mM HEPES-KOH pH 7.4, 75 mM KCl, 75 mM NaCl, ½ tablet/50 ml Protease Inhibitor, was induced by the addition 5 µM of heparin low molecular weight Impact of FibrilPaint peptides was assessed by adding 0.02, 0.2 or 2 µM of peptide.

Monitoring TauRD aggregation by thioflavin T (ThT) fluorescence

For monitoring the aggregation of TauRD by fluorescence, ThT was added to the aggregation mixture before addition of heparin. We used 45 µM ThT as standard condition, unless stated otherwise. unless stated otherwise. Fluorescence spectra were recorder every 5 minutes, at 37ºC and shaking 1 minutes at 600 rpm before recording, for 24 hours in CLARIOstar® Plus fluorescence plate reader.

Amyloid-β (1-42) expression, purtification and fibril preparation

Amyloid-beta peptide 1-42 (Aβ42) was purchased from Sigma-Aldrich (A9810). Monomers were solubilized in PBS pH 7.4, according to established protocols (11). Aliquots of 10 µM peptide were stored at -20 °C. Monomers were freshly thawed prior to experiments.

Aβ42 fibrils were prepared by quiescent incubation at 37 °C for 20 hours in presence of 0.02 w/v% NaN3. After 4 hours at room temperature (21 °C), we verified the presence of Aβ42 aggregates using AFM. AFM samples were prepared by adsorbing 10 µl of 5 times diluted sample onto mica (Muscovite mica, V 1 quality, EMS) for 45 minutes, after which the mica sheets were carefully washed with milliQ 4 times, covered and left to dry overnight. The Aβ42 fibril samples were stored at room temperature (21 °C).

α-Synuclein fibril formation

Monomeric α-Syn was thawed, supplemented with NaCl and NaN3 and diluted to final concentrations of 50 µM α-Syn, 10 mM Tris-HCl (pH 7.4), 10 mM NaCl

and 0.02 w/v% NaN3. Samples were incubated for 17 days at 37 °C while shaking at 500 rpm. To verify α-Syn aggregation, the samples were centrifuged for 30 minutes at 17000 g. The monomer residual concentration was measured to be 15 µM using UV-Vis absorption (NanoDrop, Thermo Fisher Scientific). The fibrils were resuspended and aliquoted. Fibrils were subsequently stored at room temperature (21 °C).

HttEx1Q44 expression, purification and and fibril preparation

We produced HttEx1Q44 with in E. Coli BL21 Rosetta 2 (Novagen), with an additional N-terminal MBP- and C-terminal His6-tag. Cells were induced, harvested, resuspended and disrupted as described for TauRD. Supernatant was filtered using a 0.22 µm polypropylene filtered and purified with an ÄKTA purifier chromatography System. Sample was loaded into a POROS 20MC affinity purification column with 50 mM HEPES-KOH pH 8.5, 100 mM KCl, eluted with a linear gradient 0-100%, 10 CV of 0.5 M imidazole, 30 mM NaCl. Fractions of interest were collected, buffer exchanged to 50 mM HEPES 150 mM NaCl, and concentrated to desired concentration using a Vivaspin column (vivaspin, MWCO 30 kDa). Protein concentration was measured using a NanoDrop™ OneC UV/Vis spectrophotometer and purity was assessed by SDS-PAGE. Protein was aliquoted and stored at – 80 oC.

HttEx1Q444 fibrils were prepared though addition of Factor Xa (FXa) at 0.1 µM to 20µM HttEx1 to cleave off the MBP-tag, followed by incubation at 37 °C for 4 hours in 25 mM HEPES-KOH pH 7.4, 75 mM KCl, 75 mM NaCl, ½ tablet/50 ml Protease Inhibitor. After 4 hours, we verified the presence of Aβ42 aggregates using microscale thermophoresis (MST, Prometheus panta). The HttEx1Qq44 fibril fibril samples were used immediately after preparation.

Fibril extraction

PHFs and SFs were extracted from grey matter of prefrontal cortex from one patient diagnosed with AD according to established protocols (3). Tissue was homogenized using a Polytron(PT 2500E, Kinematica AG) on max speed in 20 % (w/v) A68 buffer, consisting of 20 mM TRIS-HCl pH 7.4, 10 mM EDTA, 1.6 M NaCl, 10% sucrose, 1 tablet/10 ml Pierce protease inhibitor, 1 tablet/10 ml phosphatase inhibitor. The homogenized sample was spun from 20 minutes, at 14000 rpm at 4 ^{o}C. Supernatant was collected, and the pellet was homogenized in 10% (w/v) A68 buffer. The homogenized was spun once more. The supernatants of both centrifugations were combined and supplied with 10% w/v Sarkosyl, and incubated for 1 hour on a rocker at room temperature. The sample was ultracentrifuged for 1 hour at 100,000 g and 4 ^{o}C. The supernatant was discarded, and the pellet was incubated overnight at 4 ^{o}C in 20 µl/0.2 g starting material of 50 mM TRIS-HCl pH 7.4. The next day, the pellet was diluted up to 1 ml of A68 buffer, and resuspended. To get rid of contamination, the sample was spun for 30 minutes at 14000 rpm, 4 ^{o}C. Supernatant was collected and spun once more for 1 hour at 100000xg, 4 ^{o}C. The pellet was resuspended in 30 µl 25 mM HEPES-KOH pH 7.4, 75 mM KCl, 75 mM NaCl, and stored at 4 ^{o}C up to a month. Presence of PHF and SF was assessed by TEM.

Narrow filaments were extracted from the grey matter of the middle frontal gyrus from one patient diagnosed with FTD (nhb 2017-019), as described in established protocols (4). Fibrils were extracted following the protocol for AD fibrils. After the

first ultracentrifugation step, the pellet was resuspended in 250 µl/1g of starting material of 50 mM Tris pH 7.5, 150 mM NaCl, 0.02% amphipol A8-35. The sample was centrifuged for 30 minutes at 3000 g and 4 °C. Pellet was discarded, and the supernatant was ultracentrifuged for 1 hour at 100000xg and 4 °C. The pellet was resuspended in 30 µl of 50 mM TRIS-HCl pH 7.4, 150 mM NaCl, and stored at 4 °C up to a month. Presence of narrow filaments was assessed by TEM.

CBD fibrils were extracted from the grey matter of the superior parietal gyrus of one patient diagnosed with CBD (nhb 2018-007), following established protocols (12). Tissue was homogenized using a Polytron (PT 2500E, Kinematica AG) on max speed in 20 % w/v 10 mM TRIS-HCl pH 7.5, 1 mM EGTA, 0.8 M NaCl, 10% sucrose. The homogenate was supplied with 2% w/v of sarkosyl and incubated for 20 minutes at 37 °C. The sample was centrifuged for 10 minutes at 20000 g, and 25 °C. The supernatant was ultracentrifuged for 20 minutes at 100000 g and 25 °C. The pellet was resuspended in 750 µl/1g starting material of 10 mM TRIS-HCl pH 7.5, 1 mM EGTA, 0.8 M NaCl, 10% sucrose, and centrifuged at 9800xg for 20 minutes. The supernatant was ultracentrifuged for 1 hour at 100,000 g. The pellet was resuspended in 25 µl/g starting material of 20 mM TRIS-HCl pH 7.4, 100 mM NaCl, and stored at 4 °C up to a month. Presence of CBD fibrils was assessed by TEM.

Brain material of FTD and CBD were obtained from the Dutch Brain Bank, project number 1369. Brain material for AD was donated by prof. J. M. Hoozemans from the VU Medical Centra Amsterdam.

Electron Microscopy

Fibril samples were diluted to concentrations between 2 µM and 20 µM, and stored in an ice bath until preparation of grids at room temperature. Copper grids with continuous carbon were glow discharged in air of 0.1 bar for 15 sec, using a current of 10 mA, before applying a sample volume of 2.0 µL that was allowed to incubate on the carbon for 60 sec. After incubation, the grid was blotted and subsequently washed twice in 4 µL of milli-Q water and stained twice in 4 µL of uranyl acetate (2w/v% in water), making sure to blot it dry before proceeding to the next droplet. The second droplet of uranyl acetate was left to incubate for 60 sec on the grid, after which it was blotted nearly entirely and left to evaporate for 5 minutes. Imaging took place on a Talos L120C transmission electron microscope from Thermo Fisher Scientific, operating at 120 kV in micro-probe mode. Using a defocus between -1.0 µm and -2.0 µm, the CETA camera recorded images at magnifications between 210 and 120.000x, corresponding to an approximate dose between 1 e/ Å^2 and 50 e/ Å^2 depending on the exact magnification.

Fibril length was quantified from negatively stained EM images using Fiji (ImageJ) software. Only fibrils entirely visible within the defined analysis area were included, with measurements of over 100 individual fibrils per condition. Length was determined directly, without relying on structural markers, except in the case of Alzheimer-derived fibrils, where the characteristic helical twists of paired helical filaments (PHFs) were used to estimate fibril length.

FibrilRuler Test: Size measurement with Flow Induced Dispersion Analysis (FIDA)

Flow Induced Dispersion Analysis (FIDA) was used to determine fibril size. The FIDA experiments were performed using a FIDA1 with a 480 nm excitation source. The mode of operation use is capillary dissociation (Capdis). In this, the capillary is equilibrated with buffer followed by injection of the sample. Only the indicator sample contains fibrils, in order to minimize stickiness to the capillary (**Table 3**).

Different timepoints preformed TauRD fibrils were diluted to a final concentration of 2 µM in 25 mM HEPES-KOH pH 7.5, 75 mM KCl, 75 mM NaCl, 0.5% pluronic, together with 200 nM of FibrilPaint1. For patient derived Tau filaments, AD, CBD or FTD fibrils were diluted to a final concentration of 2 µM in 20 mM TRIS-HCl pH 7.4, 100 mM NaCl, 0.5 % pluronic (for AD and CBD), or 50 mM TRIS-HCl pH 7.4, 150 mM NaCl, 0.5 % pluronic (for FTD).

Data points were analysed using FIDAbio software, version 2.7. Samples were autoanalysed with a baseline-correction at 1 minute, and three species were distinguished. Only the largest species was plotted in all graphs.

Microscale thermophoresis binding analysis

Binding between FibrilPaint1 and TauRD monomers and fibrils was analyzed using Monolith (NanoTemper Technologies) Microscale thermophoresis system (MST). Thermophoresis was monitored at a concentration of 50 nM of FibrilPaint1 and a dilution series of the sample of interest, in 25 mM HEPES-KOH pH 7.5, 75 mM KCl, 75 mM NaCl. Samples were transferred to premium capillaries (NanoTemper Technologies) and measurements were performed at 37 ^{o}C, with medium blue LED power and at MST infrared laser power of 50% to induce thermophoretic motion. The infrared laser was switched on 1 s after the start of the measurement for a 20 s period. Datapoints used were the MST response at 5 seconds.

Calculation of fibril length from R_h

For a perfectly spherical particle, the R_h is identical to the radius of the sphere. For fibrils, the shape can be approximated as a cylinder. When the length is at least five times as big as the radius (L/R>5), we can apply the Equation 1 (13, 14):

Equation 1:

$$L = 2\ R_h\ S$$

$$s = \ln\left(\frac{L}{R} + \left(\frac{L^2}{R^2} + 1\right)^{\frac{1}{2}}\right) - \frac{R}{L}\left(\frac{L^2}{R^2} + 1\right)^{\frac{1}{2}} + \frac{R}{L}$$

Using equation 1, where L is the length and R the radius of the analysed particle, the mean length of the fibrils as function of R_h can be calculated.

As a model, PHF Fibril layers were stacked on top of each other using PyMOL software. 10 structures were created with layers from 1 to 1000 layers. Resulting structures were analysed for their R_h using FIDAbio hydrodynamic radii predictor.

The R_h were plotted against their layers and analysed with the two-phase association model of GraphPad Prism software v10.4. The resulting mathematical equation can be used to convert the R_h to fibril layers, or the R_h can therefore be converted to fibril length, as one layer of fibril is 0.47 nm long.

References

1. D. Hagemans, I. A. M. van Belzen, T. Morán Luengo, S. G. D. Rüdiger, A script to highlight hydrophobicity and charge on protein surfaces. *Front. Mol. Biosci.* **2**, 56 (2015).
2. W. Zhang *et al.*, Heparin-induced tau filaments are polymorphic and differ from those in Alzheimer's and Pick's diseases. *eLife* **8**, 10.7554/eLife.43584 (2019).
3. A. W. P. Fitzpatrick *et al.*, Cryo-EM structures of tau filaments from Alzheimer's disease. *Nature* **547**, 185-190 (2017).
4. B. Falcon *et al.*, Structures of filaments from Pick's disease reveal a novel tau protein fold. *Nature* **561**, 137-140 (2018).
5. B. Falcon *et al.*, Novel tau filament fold in chronic traumatic encephalopathy encloses hydrophobic molecules. *Nature* **568**, 420-423 (2019).
6. E. Conti, N. P. Franks, P. Brick, Crystal structure of firefly luciferase throws light on a superfamily of adenylate-forming enzymes. *Structure (London, England : 1993)* **4**, 287-298 (1996).
7. R. Guerrero-Ferreira *et al.*, Cryo-EM structure of alpha-synuclein fibrils. *eLife* **7**, e36402 (2018).
8. B. Li *et al.*, Cryo-EM of full-length α-synuclein reveals fibril polymorphs with a common structural kernel. *Nat. Commun.* **9**, 3609 (2018).
9. L. Gremer *et al.*, Fibril structure of amyloid-ss(1-42) by cryoelectron microscopy. *Science (New York, N.Y.)* eaao2825 [pii] (2017).
10. M. Bagherpoor Helabad *et al.*, Integrative determination of atomic structure of mutant huntingtin exon 1 fibrils implicated in Huntington disease. *Nat Commun* **15**, 10793 (2024).
11. A. Vandersteen *et al.*, A comparative analysis of the aggregation behavior of amyloid-β peptide variants. *FEBS Lett.* **586**, 4088-4093 (2012).
12. W. Zhang *et al.*, Novel tau filament fold in corticobasal degeneration. *Nature* **580**, 283-287 (2020).
13. L. Z. He, B. Niemeyer, A Novel Correlation for Protein Diffusion Coefficients Based on Molecular Weight and Radius of Gyration. *Biotechnology Progress* **19**, 544-548 (2003).
14. T. Yoshizaki, H. Yamakawa, Dynamics of spheroid-cylindrical molecules in dilute solution. *J. Chem. Phys.* **72**, 57-69 (1980).
